# War Injuries and Neuroses of Otological Interest

**Published:** 1918

**Authors:** 


### OPHTHALMOLOGY AND OTO-LARYNGOLOGY

### *War Injuries and Neuroses of Otological Interest.* By Dr. C. E. JONES-PHILLIPSON, *Journ. Laryn., Rhin. and Otol.*

Jones-Phillipson, while in charge of the ear cases for the Third Army of the British troops, made notes of 1172 cases. He analysed 100 cases of war injury of the ear, all of which were seen within 24 to 36 hours of their coming out of the trenches. There were 31 cases of laceration of the external ear : in the antero-inferior quadrant in 20; in the postero-inferior quadrant in 6; in the inferior quadrant in 1; in the umbo in 2; in the posterior quadrant across the two in 1. The lacerations usually healed well and quickly.

He made notes of 100 cases who definitely attributed their deafness, or increased deafness, to the effects of " guns, " " explosions, " " bombardment, " and " being buried ". 70 per cent of them showed pre-existing ear, throat and nose conditions : defects due to otitis media suppuration, 34; defects due to C. D. C., 22; defects due to nose and nasopharynx, 17.

The patients with ear injuries complained of the following symptoms, in order of frequency :

(1) Deafness, increased deafness, " dulness in ears. "

(2) Noises in great variety (singing, buzzing, hissing, straining, thumping, bells, throbbing, ticking).

(3) Giddiness, dizziness, " dazed ".

(4) Pain — soon passing off.

(5) Bleeding, at the time, or noticed soon after.

(6) Staggering gait, inability to walk, unconscious, dumbness, blindness.

Deafness was very marked soon after the explosion, always most on the side exposed to the full force; when the shell burst in front or behind a patient, both ears were affected. Patients who had been buried seemed to have suffered more than others. The initial degree of deafness following the concussion soon passed off. The noises were often noticed only later, after the first degree of deafness had passed off. It was not often that increase in deafness was stated; as a rule, only the addition of noises to the previous condition. When the hearing showed further marked improvement, the persistence of the noises was a common complaint. Some patients reported being unconscious for from one to two hours; then feeling in a dazed condition, inability to walk unassisted, staggering gait; others that they lost the power to speak, or could not see. All these maximum conditions appear to pass off quickly; the lesser conditions, giddiness and dizziness, were complained of for seven to ten to fourteen days — anyway while under observation. These cases showed a horizontal or rotatory nystagmus, or turning eyes to the right or left. It must be noted whether both right and left sides were likely to have been affected at the time, or right or left only. It will be noticed that when both sides are affected there was a nystagmus to the right and left; when one side was affected there was a nystagmus to the opposite side.

From his study of this series of cases Jones-Phillipson concludes that shell-concussion deafness is to a large extent temporary and curable. He thinks that shell-concussion deafness is probably due to three contributory factors:

(1) Cerebral concussion.

(2) Overstrain and fatigue of the organ of Corti; the former being due to violent oscillations of the perilymph communicated to the organ of Corti, and the latter to continuous violent noises or explosions at close quarters.

(3) Temporary or permanent disorganization of the conductive apparatus.

The prognosis depends on the recovery of these parts.

(1) The patient experienced a great shock, and was, in many instances, buried. He became suddenly deaf, and often dumb also. In other cases, he could not see, or had paresis of arm, leg, or both legs. Here the higher centres were temporarily involved. One watched the almost sudden improvement in hearing in a few days as shock passed off, and the disappearance of nervous symptoms generally when the patient was removed from the firing line.

(2) A portion of deafness remains to be more or less slowly recovered from by the return of the internal ear to a normal or nearly normal condition.

(3) Structural damage must leave a permanent imperfection of function — a ruptured membrane; a dislocation, partial or complete, of the small bones from one another, or the stretching of their attachments to the tympanic wall.

